# Machine learning based biomarker discovery for chronic kidney disease–mineral and bone disorder (CKD-MBD)

**DOI:** 10.1186/s12911-024-02421-6

**Published:** 2024-02-05

**Authors:** Yuting Li, Yukuan Lou, Man Liu, Siyi Chen, Peng Tan, Xiang Li, Huaixin Sun, Weixin Kong, Suhua Zhang, Xiang Shao

**Affiliations:** 1 Geriatrics Department, Suzhou Kowloon Hospital, Shanghai Jiao Tong University School of Medicine, Suzhou, China; 2 Hemodialysis Department, Suzhou Kowloon Hospital, Shanghai Jiao Tong University School of Medicine, Wan Shen St. 118, Suzhou, Jiangsu 215028 China; 3 School of Health Science and Engineering, University of Shanghai for Science and Technology, Shanghai, China

**Keywords:** CKD-MBD, Biomarker, Machine learning, Calcium, Hyperphosphatemia, PTH

## Abstract

**Introduction:**

Chronic kidney disease-mineral and bone disorder (CKD-MBD) is characterized by bone abnormalities, vascular calcification, and some other complications. Although there are diagnostic criteria for CKD-MBD, in situations when conducting target feature examining are unavailable, there is a need to investigate and discover alternative biochemical criteria that are easy to obtain. Moreover, studying the correlations between the newly discovered biomarkers and the existing ones may provide insights into the underlying molecular mechanisms of CKD-MBD.

**Methods:**

We collected a cohort of 116 individuals, consisting of three subtypes of CKD-MBD: calcium abnormality, phosphorus abnormality, and PTH abnormality. To identify the best biomarker panel for discrimination, we conducted six machine learning prediction methods and employed a sequential forward feature selection approach for each subtype. Additionally, we collected a separate prospective cohort of 114 samples to validate the discriminative power of the trained prediction models.

**Results:**

Using machine learning under cross validation setting, the feature selection method selected a concise biomarker panel for each CKD-MBD subtype as well as for the general one. Using the consensus of these features, best area under ROC curve reached up to 0.95 for the training dataset and 0.74 for the perspective dataset, respectively.

**Discussion/Conclusion:**

For the first time, we utilized machine learning methods to analyze biochemical criteria associated with CKD-MBD. Our aim was to identify alternative biomarkers that could serve not only as early detection indicators for CKD-MBD, but also as potential candidates for studying the underlying molecular mechanisms of the condition.

**Supplementary Information:**

The online version contains supplementary material available at 10.1186/s12911-024-02421-6.

## Introduction

Chronic kidney disease-mineral and bone disorder (CKD-MBD) is one of the many complications associated with chronic kidney disease. It represents a systemic disorder of mineral and bone metabolism due to CKD manifested by either one or a combination of the following: abnormalities of calcium, phosphorus, parathyroid hormone (PTH), or vitamin D metabolism; abnormalities in bone turnover, mineralization, volume, linear growth, or strength; or vascular and other soft tissue calcification [1, 2]. CKD-MBD is associated with adverse outcomes including cardiovascular disease and mortality [3], and the increased awareness of the potential role played by mineral and bone disorder in the appearance of cardiovascular disease in renal patients has encouraged research efforts aimed at discovering possible pathogenic links [4]. Accordingly, the diagnostic significance of the classic bone markers of mineral disorders and of the new markers in the setting of chronic kidney disease-mineral and bone disorders (CKD-MBD) needs to be re-evaluated along with increasing information [4]. The maintenance of calcium and phosphate homeostasis is essential to an individual’s health because Ca and P are essential to many vital physiological processes. Through assisting intestinal absorption, bone mineralization/demineralization, and renal excretion/reabsorption of both ions, several organs contribute to the meticulous balancing and regulation of calcium and phosphate homeostasis, and the regulation of these processes occurs by a number of hormones [5]. The biologically active forms of vitamin D, also known as dihydroxyvitamin D3 or calcitriol, PTH, and calcitonin have been studied extensively in this regard, and fibroblast growth factor 23 (FGF23) and klotho were identified as new players essential to the regulation of calcium and phosphate homeostasis, as recent studies demonstrated [6]. In mainland China, according to the CKD-MBD Diagnosis Guide (guide.medlive.cn), calcium balance, phosphate balance, and PTH are the three key biochemical criteria for CKD-MBD diagnosis.

In the context of calcium balance, the actions of PTH and calcitriol play a crucial role in regulating serum calcium concentrations. Calcium is essential for numerous metabolic processes, and its concentration is normally maintained within a healthy range. However, in the late stages of CKD, serum calcium levels may decrease to some extent. This poses a challenge when considering calcium as a potential uremic toxin, as the calcium balance in CKD is not accurately reflected by serum calcium levels [7]. Despite ongoing controversy, several studies have provided evidence indicating that calcium loading plays a significant role in cardiovascular disease in CKD [7]. Interestingly, even though high calcium levels are often only weak predictors of adverse outcomes, this may be attributed to the weak correlation between serum calcium balance and calcium levels. Nonetheless, exposure to excess calcium may still pose a cardiovascular risk [8–10].

In the context of phosphate balance, inorganic phosphorus plays a vital role in various biological functions, including energy exchange, the production and function of cell membranes, and intracellular signal transduction. The regulation of bone formation and resorption, nutrient absorption, renal excretion, and equilibration with intracellular stores helps to maintain serum phosphate levels within the physiological range in healthy individuals. Due to the kidneys’ essential role in metabolism, it is well-known that phosphate balance is disrupted early in CKD. However, research has not revealed an increase in total-body phosphate load, at least not in predialysis CKD patients, although it is reasonable to infer that phosphate retention is common in CKD [11, 12]. Hyperphosphatemia has been indicated as a late marker of a disturbed phosphate metabolism [5]. When collaborating with calcium, phosphate can enhance calcification, particularly in vascular smooth muscle cells (VSMCs), during the formation of hydroxyapatite nanocrystals. This process involves vesicle release, osteochondrocytic differentiation, apoptosis, and perturbation of calcification inhibitor levels [13–18]. Apart from the detrimental effects of high phosphate levels on VSMCs, studies have demonstrated that endothelial function can be directly affected by elevated phosphate levels both in vitro and in vivo [19]. Moreover, hyperphosphatemia may accelerate aging, as observed in individuals with CKD [20].

Parathyroid hormone (PTH) is a single chain polypeptide of certain amino acid long that is constantly secreted at low rate by parathyroid; its secretion is usually up-regulated in response to reductions in serum calcium concentration. The entire PTH molecule, usually 84 amino acids in length, is also known as biointact or whole PTH, which is to be differentiated from other smaller PTH, known as small-molecular-weight carboxy-terminal fragments (C-PTH); the C-PTH are usually present in plasma. The hormonal fragments arise from metabolism of 1–84 PTH by peripheral organs as well as from secretion of C-PTH fragments from the parathyroid glands [21]. The pathogenesis of several CKD complications, including dyslipidemia, hypertension, carbohydrate intolerance, and peripheral neuropathy, all involves PTH [22]. PTH causes intracellular calcium increase in many cell types except for VSMCs. Both an increased influx and a decreased efflux from the cell can cause the increase in cytosolic calcium levels [12]. High PTH levels inhibit mitochondrial activities, down-regulate phosphorylation, and uncouple oxidative phosphorylation in isolated heart mitochondria, which causes adverse effects on the myocardium [23]. PTH up-regulation was also reported to be correlated with of left ventricular myocardial function and cardiac hypertrophy cardiac fibrosis abnormalities in hemodialysis patients [24, 25]. It is also worth noting that vascular calcification may due to either high or low PTH levels [26, 27], and that bone remodeling can be regulated by PTH [28].

We recognize the significant roles of calcium, phosphate, and PTH in diagnosing CKD-MBD. However, in situations where it is not feasible to measure these three features, it is crucial to explore alternative biochemical criteria that are easily obtainable. Such alternative indicators could serve as early biomarkers for detecting CKD-MBD and offer new possibilities for studying the molecular mechanisms of the condition. Machine learning-based approaches have proven effective in discovering biomarkers [29–32] in various biological and clinical contexts over the past decade [33–38]. Surprisingly, there has been no exploration of these approaches in the field of CKD-MBD. Hence, this study aims to utilize machine learning-based feature selection techniques to identify alternative CKD-MBD biomarkers.

## Materials and methods

### Data collection & curation

We collected 116 patients’ basic demographic variables (gender, age, exam date) and 86 blood biochemical criteria (features). All the patients agreed and signed the Informed Consent Form. There are 70 male and 46 female patients in the 116 cohort, with average age 60.9 and standard deviation 12.9. After removing features with > 20% missing values across the cohort, we finally obtained a 116 patient by 65 feature training dataset. Following the same procedure, we again collected and curated a 114-patient cohort to serve as standalone validation set.

### CKD-MBD classification

Within the above 65 features, three features, i.e., Calcium, Phosphorus and PTH are used to classify a patient to one of the five groups: CKDMBD-Cal, CKDMBD-Phos, CKDMBD-PTH, CKDMBD-Any and Health Control. Specifically, patients with Calcium > 2.5 or Calcium < 2.1 are considered CKDMBD-Cal; patients with Phosphorus > 1.45 or Phosphorus < 0.78 are considered CKDMBD-Phos; patients with PTH > 600 or PTH < 100 are considered CKDMBD-PTH; patients with at least one of the CKDMBD-Cal, CKDMBD-Phos and CKDMBD-PTH are considered CKDMBD-Any. We thus curated four training datasets of these four types. CKDMBD-Cal dataset contains 57 Calcium abnormal patients and 59 controls (note: controls are not necessarily health individuals and may belong to other CKDMBD types); CKDMBD-Phos dataset contains 96 Phosphorus abnormal patients and 20 controls; CKDMBD-PTH dataset contains 30 PTH abnormal patients and 86 controls; CKDMBD-Any dataset contains 108 any-type abnormal patients and 8 controls.

### Biomarker identification

We adopted a sequential forward feature selection algorithm to select biomarkers for each of the four classification/prediction sub-tasks, based on the six machine learning algorithms introduced below and a leave-one-out cross validation train-test framework. Specifically, we first calculate the F-Test score for each feature and rank the features descending according to the score. The F-Test score measures inter-class difference divided by intra-class difference. After ranking, all the features are sorted according to their discriminatory power. Then the first ranked feature was then adopted into a feature pool as seed and classifications are performed under leave-one-out cross validation. The area under ROC curve (AUC) was then calculated and recorded to measure the classification performance of this seed feature. Then the second ranked feature was added to the feature pool and the process was repeated again to obtain a new AUC. If the new AUC was greater than the old one, the second ranked feature was kept in the feature pool, otherwise it was discarded. Likewise, the process was repeated for all the following features to obtain a final selected feature pool.

### Machine learning methods

Six widely adopted Machine Learning (ML) algorithms were employed as classifiers in the feature selection and the finally classification procedure: K-Nearest Neighbor (KNN), Logistic Regression (LR), Linear Regression, Support Vector Regression (SVR), Deep Neural Network (DNN) and Random Forest (RF). The hyper-parameters of each ML method were tuned via trial and error to achieve best LOO cross validation results. Specifically, K was set to 1 in KNN after testing K = [1, 2, 3, 4, 5, 10]; ‘binomial’, and ‘logit’ for ‘link/distance’ was applied in Logistic Regression; polynomial kernel with ‘polynomial = 1’ was used in the Support Vector Regression; ‘Hidden layer number = 5’ was adopted for DNN after trying hidden layer number = [1, 3, 5, 7, 10, 15]; ‘tree number’ was set to 100 for Random Forest after trying tree number = [10, 50, 100, 200, 500].

## Results

### Correlation analysis

**Fig. 1:**
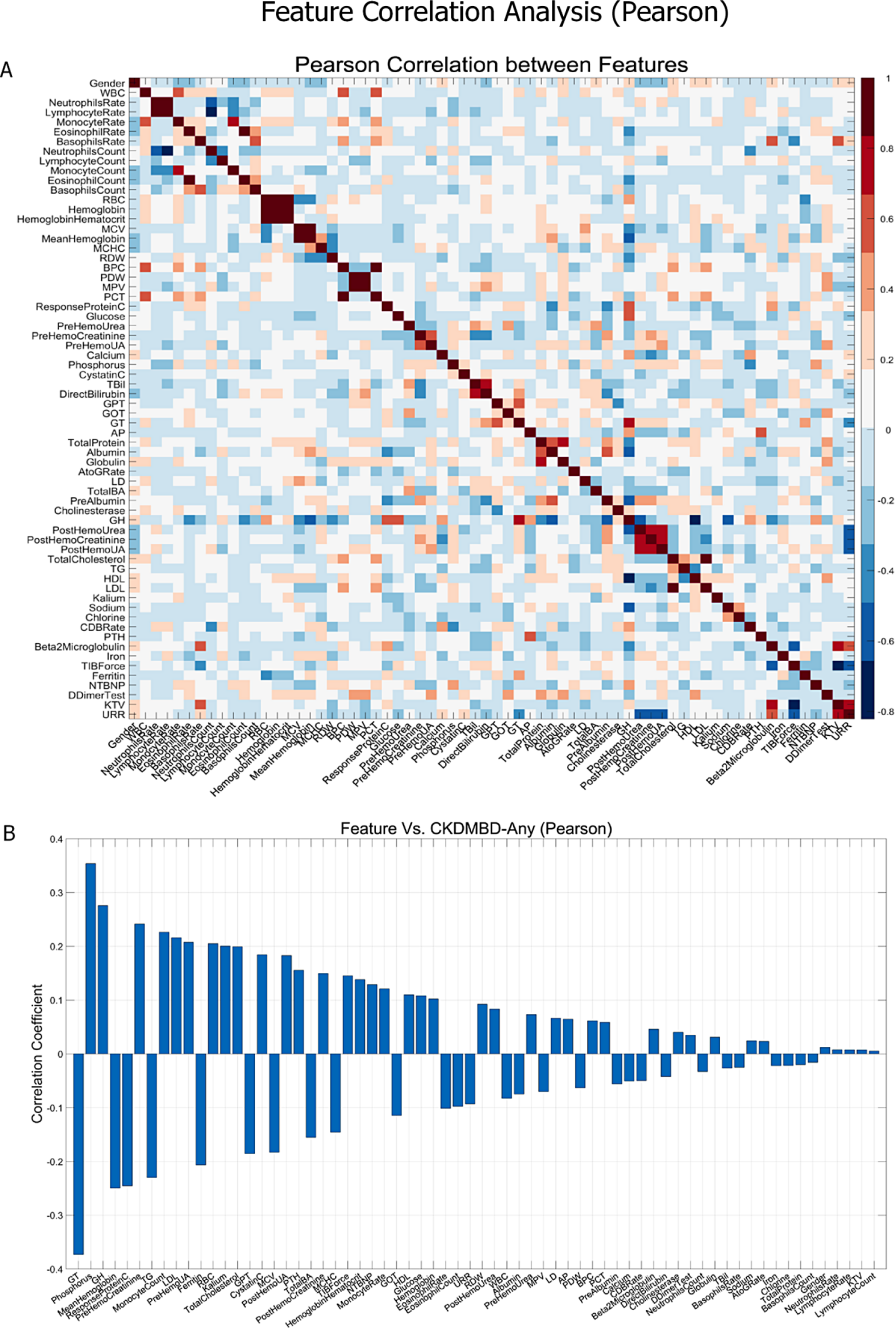
The Pearson correlation coefficient analyses. (**A**) The Pearson correlation coefficients between the 65 features. (**B**) The all-to-label Pearson correlation coefficient for CKDMBD-Any

**Fig. 2:**
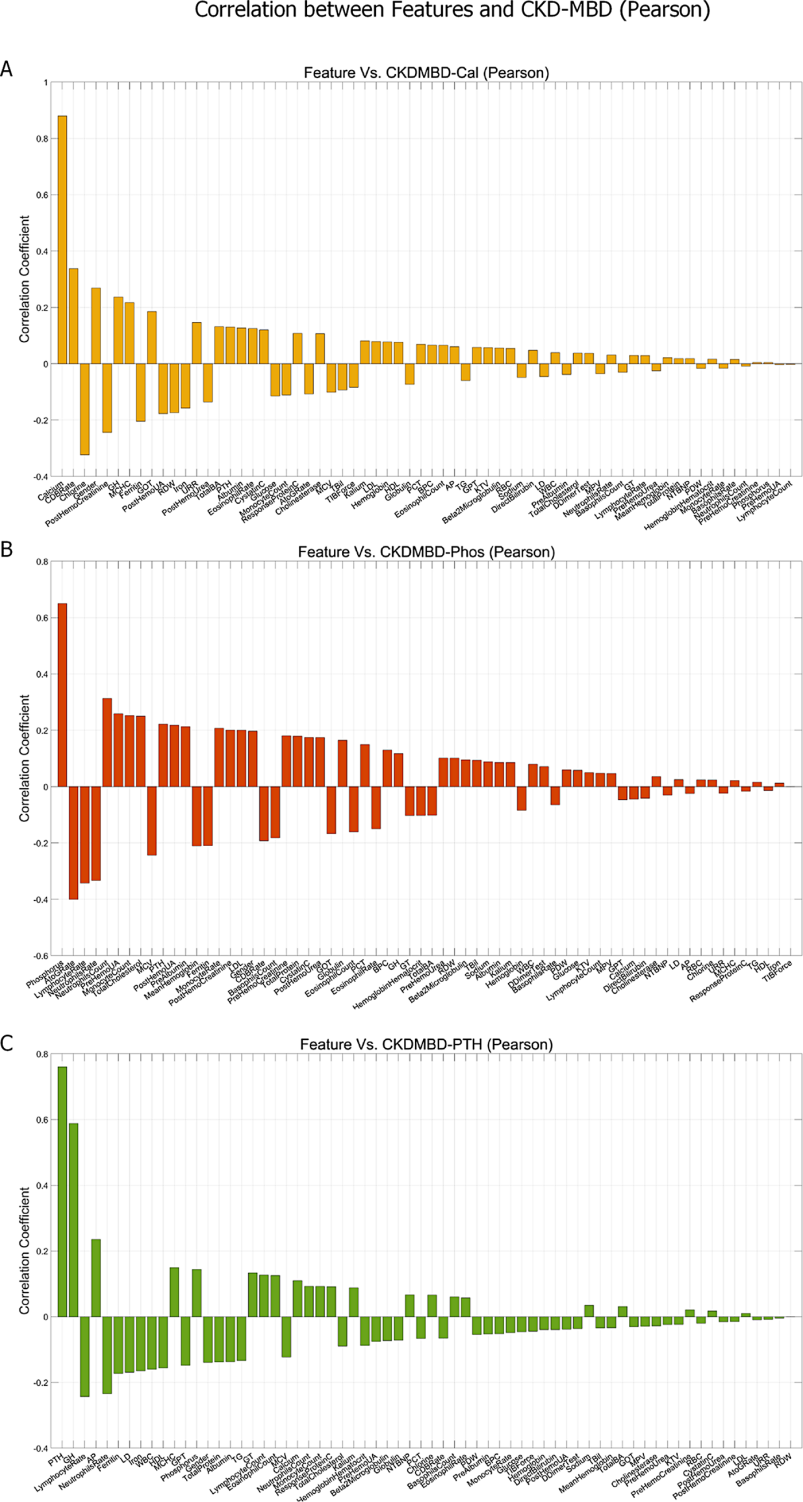
The all-to-label Pearson correlation coefficient analyses for (**A**) CKDMBD-Cal (**A**), (**B**) CKDMBD-Phos, and (**C**) CKDMBD-PTH

To examine correlations between all features, we computed the Pearson and Spearman correlation coefficients between all the 65 features (shown in Fig. 1-A and Fig. S1-A). For all the 65 × 65 pairs of correlations, the average absolute Pearson correlation is 0.127 with standard deviation 0.124, and the average absolute Spearman correlation is 0.159 with standard deviation 0.195. According to the distribution of the inter-feature correlations, the features neither belong to one big cluster nor totally independent to each other, making feature selection necessary and suitable in such scenario. To evaluate the contributions of each individual features to the four classes of CKD-MBD, we also computed the Pearson and Spearman correlation coefficients between all the features with the CKDMBD-Cal, CKDMBD-Phos, CKDMBD-PTH and CKDMBD-Any, respectively (shown in Fig. 1-B, Fig. S1-B, Fig. 2 and Fig. S2). Note that the three CKD-MBD labeling features, i.e., Calcium, Phosphorus and PTH, are not excluded and the top contributing feature is thus these three features respectively. As the correlation analyses demonstrate, most features are weakly correlated to the CKD-MBD labels, which again makes feature selection necessary and challenging.

### Feature selection

**Fig. 3:**
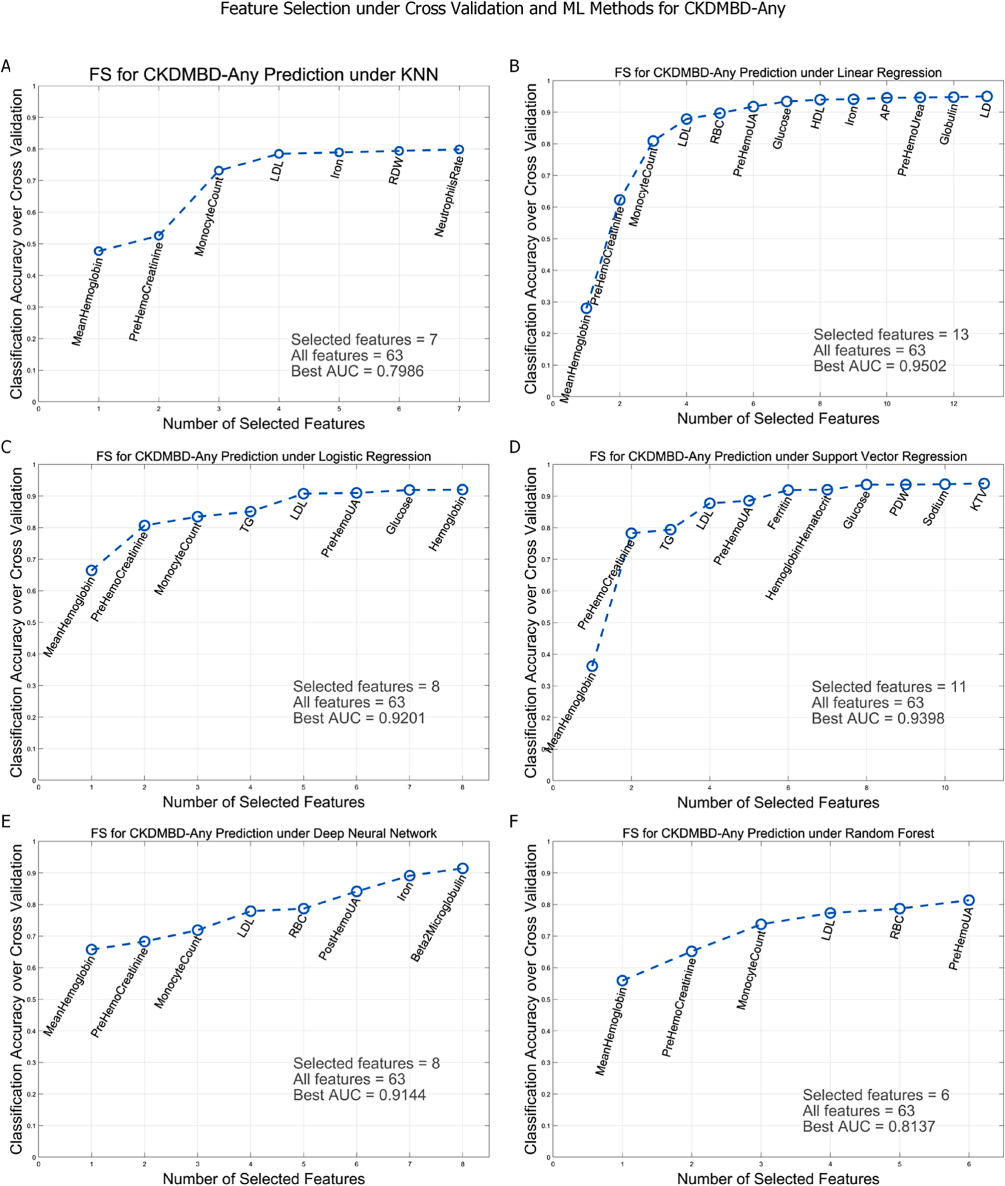
In CKDMBD-Any, the features sequentially selected by the six machine learning algorithms under leave-one-out cross validation and the corresponding AUC’s. (**A**) KNN classifier. (**B**) Linear Regression. (**C**) Logistic Regression. (**D**) Support Vector Regression. (**E**) Deep Neural Network. (**F**) Random Forest

For CKDMBD-Any, under the leave-one-out cross validation based sequential forward feature selection approach, the six machine learning methods, i.e., KNN, Linear Regression, Logistic Regression, Support Vector Regression, Deep Neural Network and Random Forest selected 7, 13, 8, 11, 8 and 6 features respectively, with maximum AUC’s 0.80, 0.95, 0.92, 0.94, 0.91 and 0.81 respectively (shown in Fig. 3). For CKDMBD-Cal, the six ML methods selected 7, 9, 8, 17, 8 and 7 features with maximum AUC’s 0.62, 0.68, 0.69, 0.99, 0.67 and 0.70 respectively (shown in Fig. S3). For CKDMBD-Phos, the six ML methods selected 4, 15, 11, 10, 6 and 5 features with maximum AUC’s 0.67, 0.80, 0.78, 0.77, 0.72 and 0.75 respectively (shown in Fig. S4). For CKDMBD-PTH, 6, 8, 10, 8, 4 and 9 features were selected with maximum AUC’s 0.75, 0.79, 0.79, 0.80, 0.72 and 0.85 respectively (shown in Fig. S5).

### Prediction based selected features

**Fig. 4:**
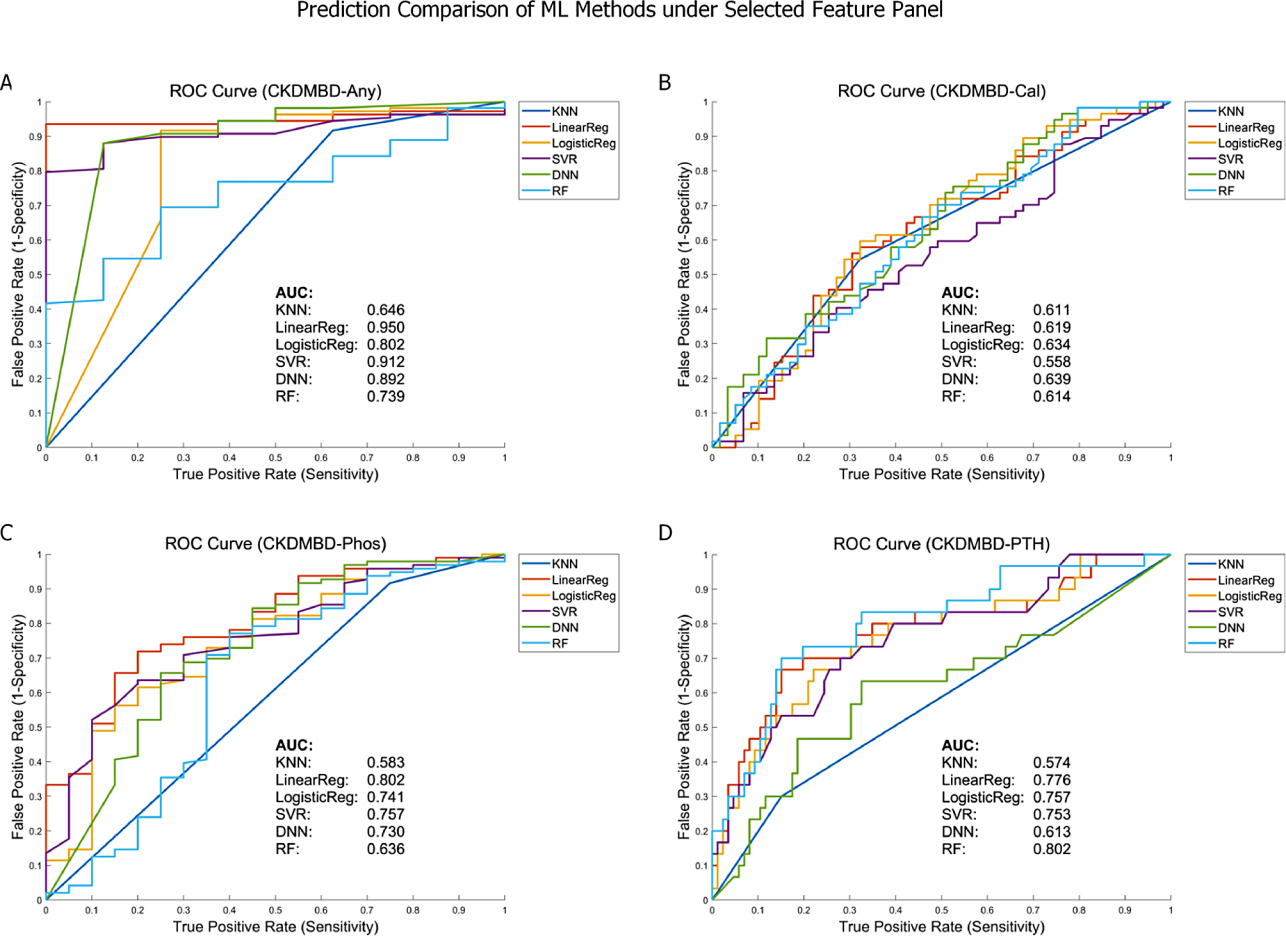
The prediction comparison of the six machine learning methods based on selected feature/biomarker panels under leave-one-out cross validation. (**A**) CKDMBD-Any. (**B**) CKDMBD-Cal. (**C**) CKDMBD-Phos. (**D**) CKDMBD-PTH

After the feature selection procedure, all the six machine learning methods jointly covered 24, 29, 26 and 23 features in CKDMBD-Cal, CKDMBD-Phos, CKDMBD-PTH and CKDMBD-Any scenario respectively. The selected features along with counts of being selected by the six ML methods are listed in Table 1 and Table S1-S3. Among these features, 9, 13, 14 and 8 features were selected by at least two machine learning methods. We adopted these features that were selected by at least two machine learning methods to compose the final biomarker panel and performed leave-one-out cross validation again using the six machine learning methods. After collecting the prediction results, the AUC curves were plotted for comparison (shown in Fig. 4). The best AUC’s are 0.91, 0.64, 0.80 and 0.80 for CKDMBD-Any, CKDMBD-Phos, CKDMBD-PTH and CKDMBD-Cal. To examine whether the predictive ability of the machine learning methods, based on the selected feature panel, can be transferred, we utilized the complete dataset of 116 patients to train the six machine learning methods. Subsequently, we tested the predictive performance on the separate dataset of 114 patients. The best AUC’s for predicting the four CKDMBD subtypes are 0.74, 0.60, 0.58 and 0.63, respectively, as shown in Fig. S6.

**Table 1:** The selected counts of the features under six machine learning methods under leave-one-out cross validation for CKDMBD-Any

Feature	Selected Count	Feature	Selected Count
LDL	6	KTV	1
MeanHemoglobin	6	HemoglobinHematocrit	1
PreHemoCreatinine	6	Hemoglobin	1
MonocyteCount	5	Sodium	1
PreHemoUA	4	AP	1
Glucose	3	Ferritin	1
RBC	3	PDW	1
Iron	3	LD	1
TG	2	NeutrophilsRate	1
Beta2Microglobulin	1	Globulin	1
PostHemoUA	1	RDW	1
HDL	1	PreHemoUrea	1

## Discussion/Conclusion

Although there exist diagnostic criteria for chronic kidney disease-mineral and bone disorder (CKD-MBD), there are often situations where measuring blood calcium, phosphate, and PTH levels is impractical, infrequent, or costly in remote locations. Even when these measurements are available, the accuracy of CKD-MBD diagnosis is not guaranteed due to the presence of false positives and false negatives. Hence, this study aimed to investigate alternative biochemical criteria associated with CKD-MBD. We initially constructed a dataset consisting of 116 samples, each characterized by 65 blood biochemical criteria, and categorized into four groups: calcium abnormality, phosphorus abnormality, PTH abnormality, and healthy. Subsequently, the dataset was restructured into four training datasets, namely CKDMBD-Cal, CKDMBD-Phos, CKDMBD-PTH, and CKDMBD-Any. Through the use of six machine learning classification methods and sequential forward feature selection techniques, we identified an optimal biomarker panel capable of discriminating between different types of CKD-MBD. By considering the consensus of these features, we achieved an impressive area under the receiver operating characteristic curve (AUC) of up to 0.91, which is practically significant. Notably, the features consistently selected by all six machine learning methods deserve attention, including KTV and GH for diagnosing CKDMBD-Cal; EosinophilCount for diagnosing CKDMBD-Phos; PostHemoCreatinine, AP, and Globulin for diagnosing CKDMBD-PTH; and PreHemoCreatinine, MeanHemoglobin, and LDL for CKDMBD-Any. The adopted machine learning methods varied in complexity, ranging from simple linear regression to advanced techniques such as deep neural networks and random forests, enabling the capture of both linear and non-linear correlations between features and outcomes. Thus far, Linear Regression, Deep Neural Network, and Random Forest are the three most consistent prediction methods in both training and validation datasets, based on the selected consensus feature panels. In real-world clinics, Linear Regression is encouraged for simple and quick prediction purpose, while DNN and RF can provide more robust and accurate predictions if sufficient computational resources are available. Further study of the feature panel that are more specifically customized for different CKD-MBD subtype prediction can be investigated so that in practice, more accurate prediction can be delivered. Although the sample size of the two separate cohorts for training and validation dataset are small, the ML method can achieve much robust prediction if more samples are included in the future.

To the best of our knowledge, this is the first study to employ machine learning approaches to explore biochemical criteria associated with different types of CKD-MBD, aiming to discover alternative biomarkers that serve not only as early detection indicators but also as candidates for in-depth molecular mechanism studies of CKD-MBD. We also emphasize the significance of easily obtainable biochemical criteria in predicting CKD-MBD when the target feature examinations are unavailable. We hope that this work advances the field of CKD-MBD research by promoting the use of machine learning techniques for identifying alternative biomarkers.

### Electronic supplementary material

Below is the link to the electronic supplementary material.

Supplementary Material 1

## Data Availability

All data generated or analyzed during this study are available upon reasonable request.
