# Supplementary material for: Machine learning based biomarker discovery for chronic kidney disease–mineral and bone disorder (CKD-MBD)
Source: BMC Med Inform Decis Mak. 2024 Feb 5;24:36. doi: 10.1186/s12911-024-02421-6 (PMC10840173; doi:10.1186/s12911-024-02421-6)
Supplement: Supplementary file 1 — Supplementary Material 1 [file 12911_2024_2421_MOESM1_ESM.docx]

***Machine Learning based biomarker discovery for chronic kidney disease–mineral and bone disorder (CKD-MBD)***

- **Supplementary Material**

Yuting Li^a,b,c^, Yukuan Lou^b,c^, Man Liu^b^, Siyi Chen^b^, Peng Tan^b^, Xiang Li^b^, Huaixin Sun^b^, Weixin Kong^b^, Suhua Zhang^b^, Xiang Shao^b^*

^a^ Geriatrics Department, Suzhou Kowloon Hospital, Shanghai Jiao Tong University School of Medicine, Suzhou, China

^b^ Hemodialysis Department, Suzhou Kowloon Hospital, Shanghai Jiao Tong University School of Medicine, Suzhou, China

^c^ School of Health Science and Engineering, University of Shanghai for Science and Technology, Shanghai, China

Short Title: CKD-MBD Biomarker Discovery

* Corresponding Authors:

Xiang Shao

Hemodialysis Department

Suzhou Kowloon Hospital, Shanghai Jiao Tong University School of Medicine

Wan Shen St. 118

Suzhou, Jiangsu, 215028, China

Tel: 15952440841, 13052407016

E-mail: 385534405@qq.com

Keywords: CKD-MBD, Biomarker, Machine Learning, Calcium, Hyperphosphatemia, PTH


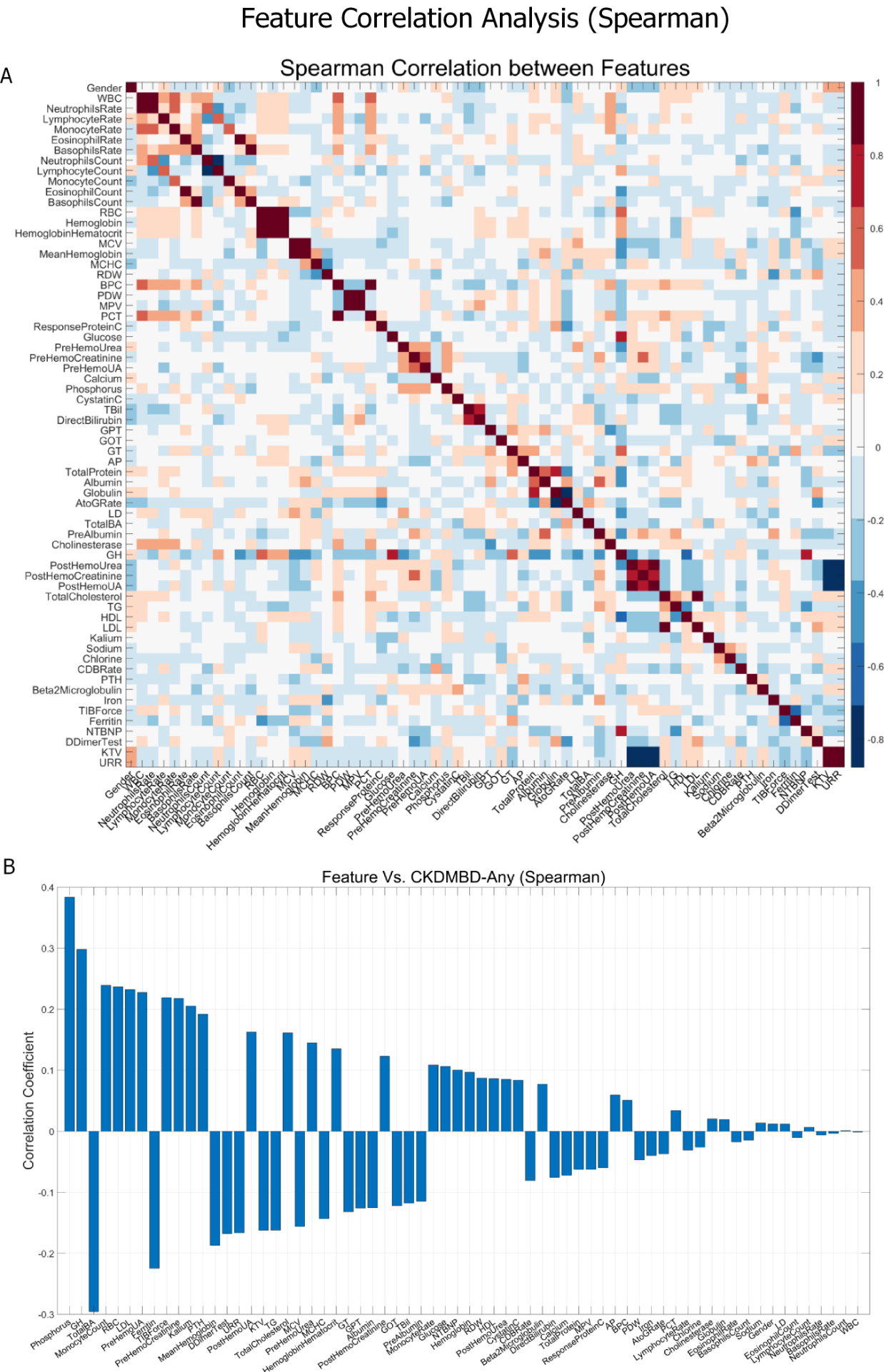


Fig. S1. The Spearman feature correlation coefficient analyses. (A) The Spearman correlation coefficients between the 66 features. (B) The feature-to-label Spearman correlation coefficient for CKDMBD-Any.


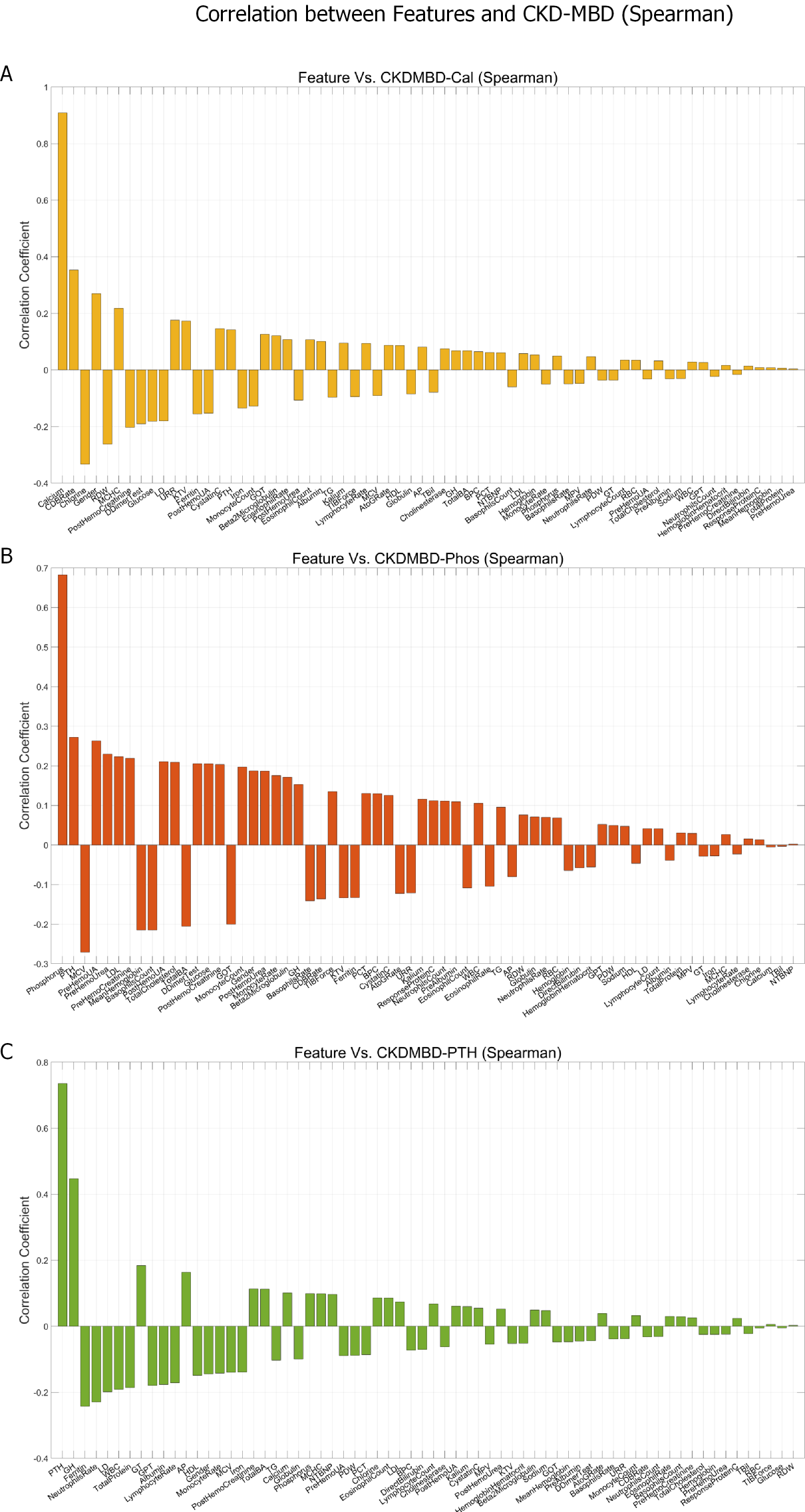


Fig. S2. The feature-to-label Spearman correlation coefficient analyses for CKDMBD-Cal (A), CKDMBD-Phos (B), and CKDMBD-PTH (C)


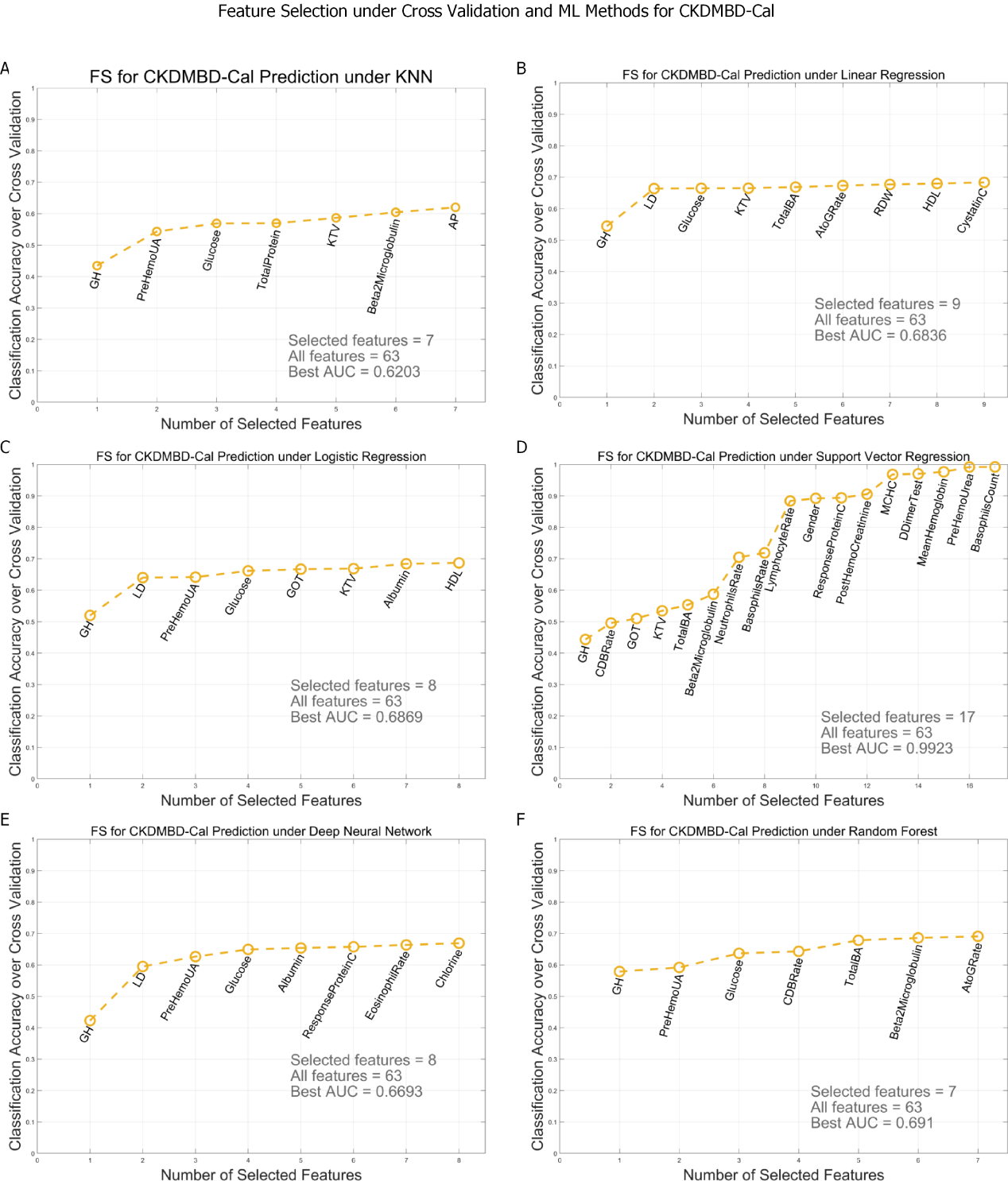


Fig. S3. In CKDMBD-Cal, the features sequentially selected by the six machine learning algorithms under leave-one-out cross validation and the corresponding AUC’s. (A) KNN classifier. (B) Linear Regression. (C) Logistic Regression. (D) Support Vector Regression. (E) Deep Neural Network. (F) Random Forest.


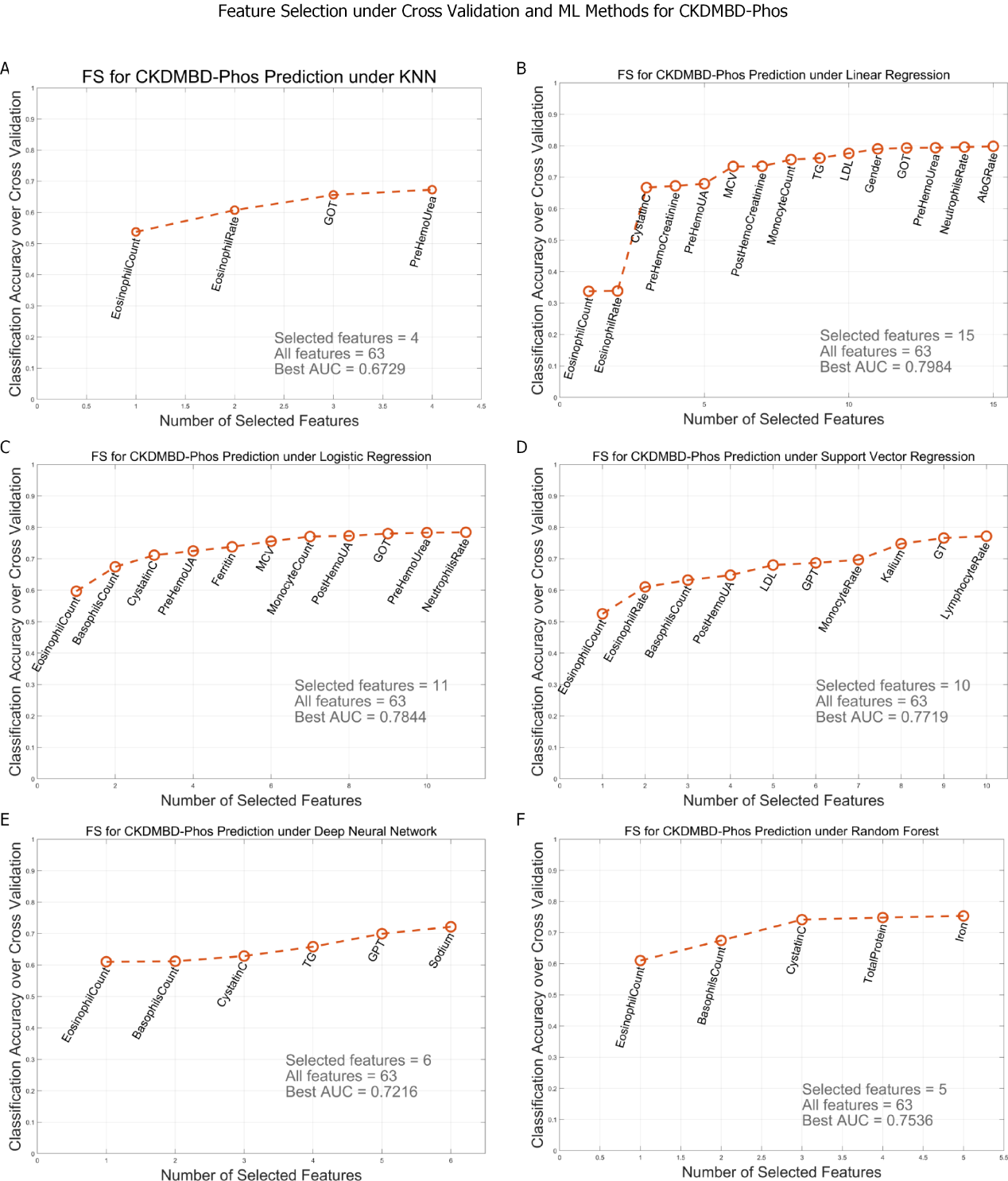


Fig. S4. In CKDMBD-Phos, the features sequentially selected by the six machine learning algorithms under leave-one-out cross validation and the corresponding AUC’s. (A). KNN classifier. (B) Linear Regression. (C) Logistic Regression. (D) Support Vector Regression. (E) Deep Neural Network. (F) Random Forest.


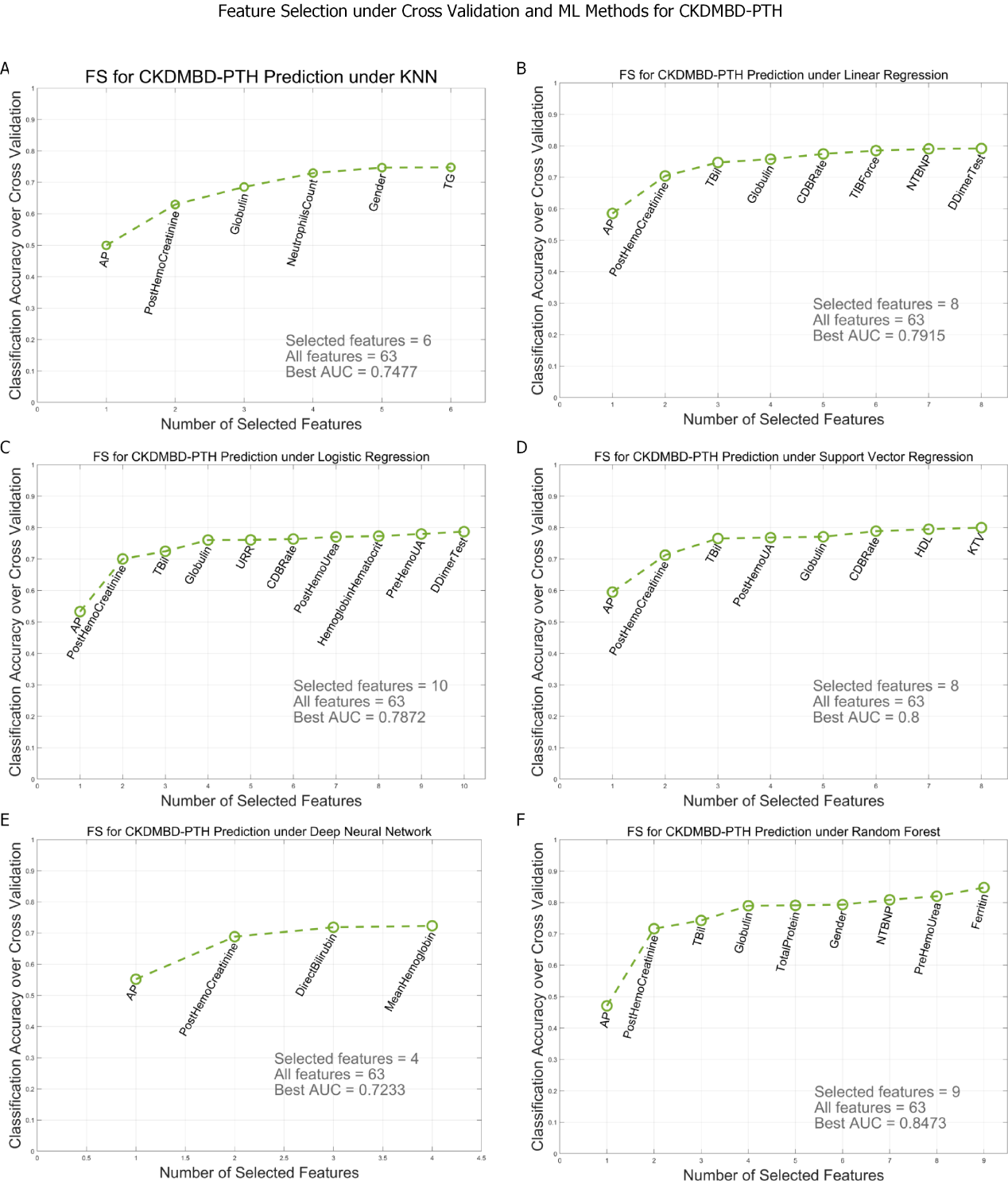


Fig. S5. In CKDMBD-PTH, the features sequentially selected by the six machine learning algorithms under leave-one-out cross validation and the corresponding AUC’s. (A) KNN classifier. (B) Linear Regression. (C) Logistic Regression. (D) Support Vector Regression. (E) Deep Neural Network. (F) Random Forest.


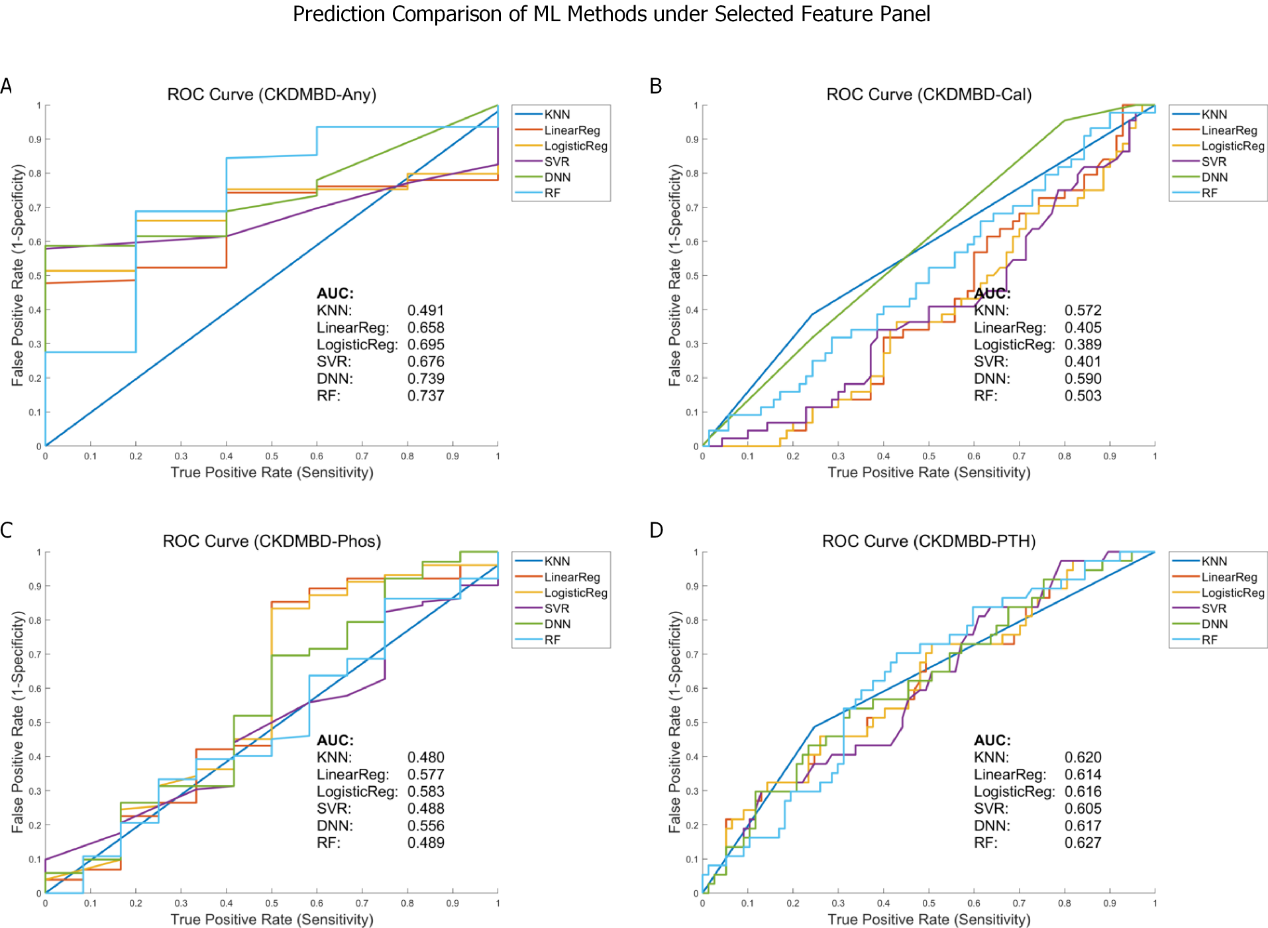


Fig. S6. The prediction comparison of the six machine learning methods trained based on selected feature/biomarker panels of the training dataset and tested for the standalone validation dataset. (A) CKDMBD-Any. (B) CKDMBD-Cal. (C) CKDMBD-Phos. (D) CKDMBD-PTH.

Table. S1. The selected counts of the features under six machine learning methods under leave-one-out cross validation for CKDMBD-Cal

| **Feature** | **Selected Count** |
| --- | --- |
| GH | 6 |
| Glucose | 5 |
| KTV | 4 |
| PreHemoUA | 4 |
| Beta2Microglobulin | 3 |
| LD | 3 |
| TotalBA | 3 |
| ResponseProteinC | 2 |
| AtoGRate | 2 |
| CDBRate | 2 |
| GOT | 2 |
| Albumin | 2 |
| HDL | 2 |
| PostHemoCreatinine | 1 |
| TotalProtein | 1 |
| Chlorine | 1 |
| NeutrophilsRate | 1 |
| BasophilsCount | 1 |
| DDimerTest | 1 |
| Gender | 1 |
| EosinophilRate | 1 |
| CystatinC | 1 |
| MeanHemoglobin | 1 |
| PreHemoUrea | 1 |
| BasophilsRate | 1 |
| AP | 1 |
| RDW | 1 |
| MCHC | 1 |
| LymphocyteRate | 1 |

Table. S2. The selected counts of the features under six machine learning methods under leave-one-out cross validation for CKDMBD-Phos

| **Feature** | **Selected Count** |
| --- | --- |
| EosinophilCount | 6 |
| BasophilsCount | 4 |
| CystatinC | 4 |
| EosinophilRate | 3 |
| PreHemoUrea | 3 |
| GOT | 3 |
| PostHemoUA | 2 |
| NeutrophilsRate | 2 |
| TG | 2 |
| PreHemoUA | 2 |
| GPT | 2 |
| MonocyteCount | 2 |
| LDL | 2 |
| MCV | 2 |
| MonocyteRate | 1 |
| Sodium | 1 |
| Kalium | 1 |
| Gender | 1 |
| Iron | 1 |
| PostHemoCreatinine | 1 |
| GT | 1 |
| TotalProtein | 1 |
| Ferritin | 1 |
| AtoGRate | 1 |
| LymphocyteRate | 1 |
| PreHemoCreatinine | 1 |

Table. S3. The selected counts of the features under six machine learning methods under leave-one-out cross validation for CKDMBD-PTH

| **Feature** | **Selected Count** |
| --- | --- |
| AP | 6 |
| PostHemoCreatinine | 6 |
| Globulin | 5 |
| TBil | 4 |
| CDBRate | 3 |
| DDimerTest | 2 |
| Gender | 2 |
| NTBNP | 2 |
| PreHemoUrea | 1 |
| DirectBilirubin | 1 |
| Ferritin | 1 |
| URR | 1 |
| HDL | 1 |
| TG | 1 |
| KTV | 1 |
| TIBForce | 1 |
| TotalProtein | 1 |
| PostHemoUrea | 1 |
| HemoglobinHematocrit | 1 |
| PostHemoUA | 1 |
| PreHemoUA | 1 |
| NeutrophilsCount | 1 |
| MeanHemoglobin | 1 |
